# CEASE Tobacco Cessation Program: Validation of Self-Rated Quit with Fagerstrom Test for Nicotine Dependence

**DOI:** 10.31586/gjcd.2025.1190

**Published:** 2025-02-07

**Authors:** Payam Sheikhattari, Rifath Ara Alam Barsha, Chidubem Egboluche, Adriana Foster, Shervin Assari

**Affiliations:** 1 Prevention Sciences Research Center, Morgan State University, Baltimore, MD, USA; 2 School of Community Health & Policy, Morgan State University, Baltimore, MD, USA; 3 Johns Hopkins University, Baltimore, MD, USA; 4 Charles R Drew University of Medicine and Science, Los Angeles, CA, USA

**Keywords:** Smoking Cessation, Underserved Populations, Low-Income Smokers, Tobacco

## Abstract

**Background:**

Despite advancements in smoking cessation interventions, few programs have demonstrated sustained effectiveness among low-income, underserved populations. The Communities Engaged and Advocating for a Smoke-free Environment (CEASE) program was developed to address this gap and support tobacco cessation in these communities. However, it remains unclear whether self-reported outcome measures in this context are in line with more objective outcome measures.

**Aims:**

This study aimed to validate self-reported quit rates using the Fagerström Test for Nicotine Dependence (FTND) as a gold standard outcome measure for evaluation of the effectiveness of the CEASE smoking cessation intervention compared to a self-help approach among low-income, underserved adult smokers.

**Methods:**

A quasi-experimental design was employed to evaluate this community-based intervention. Although participants were initially assigned to three groups, this report focuses on two arms that show the major difference in the efficacy of the program: (1) the self-help group (reference; Arm 1) and (2) the in-person CEASE group (Arm 2). Outcomes included successful quitting, assessed through self-reports, and changes in FTND scores. To examine the concordance between these measures, we tested whether changes in FTND scores fully explained the relationship between the intervention and self-reported quitting. Potential confounders included demographic, socioeconomic, and health-related variables. Data were analyzed using regression and structural equation modeling (SEM).

**Results:**

The majority of participants were Black Americans, followed by White individuals and those of other racial backgrounds. The CEASE intervention (Arm 2) demonstrated effectiveness in reducing nicotine dependence (measured by FTND) and increasing self-reported quit rates compared to the self-help group. Importantly, changes in FTND scores fully explained the effect of the CEASE intervention on self-reported quitting, highlighting the program’s impact on addiction severity.

**Conclusion:**

Successful quitting measured using self-report is in line with the decline in nicotine addiction severity among low-income racial minority populations. CEASE holds promise as a scalable solution to address smoking disparities in underserved communities.

## 1. Background

Smoking remains the leading cause of preventable death in the United States, responsible for over 480,000 premature deaths annually, including more than 41,000 due to secondhand smoke exposure [1,2]. This substantial public health burden underscores the need for effective smoking cessation interventions to reduce tobacco use.

Over the past few decades, smoking prevalence in the U.S. has decreased significantly, from 42.4% in 1965 to 15.1% in 2015 [2]. This progress is largely attributed to advances in smoking cessation strategies, such as nicotine replacement therapy (NRT), which helps reduce dependence by providing controlled doses of nicotine through products like patches and gum [3]. Other contributing factors include pharmacological aids such as varenicline and bupropion, behavioral counseling, anti-smoking campaigns, and tobacco control policies like clean indoor air laws [2,4,5]. Despite these advancements, significant health disparities in tobacco use persist. In Maryland, approximately 15.1% of adults smoke, with even higher rates observed in specific low-income areas of Baltimore. Surveys conducted through the Communities Engaged and Advocating for a Smoke-free Environment (CEASE) program reported smoking rates of 27% among parents of school-aged children and up to 75% among adults in certain community settings [6].

To address these disparities, culturally tailored smoking cessation programs that incorporate education, counseling, and social support are essential. Evidence suggests that smokers who are well-informed, motivated, and adequately prepared are more likely to quit successfully [7]. Social support, in particular, plays a critical role in overcoming behavioral triggers and environmental cues associated with smoking [8]. However, underserved communities face unique barriers to quitting, including limited access to evidence-based interventions. In the U.S., while 40% of smokers attempt to quit each year, many rely solely on willpower, leading to high relapse rates within six months [7,9]. Racial and ethnic minorities, individuals with low incomes, and those with lower educational attainment are even less likely to access effective interventions, such as counseling, quit lines, or medications [10]. Moreover, strict eligibility criteria for clinical trials often exclude marginalized populations, reducing the real-world applicability of many cessation programs.

The CEASE initiative, launched in 2007 as a Community-Based Participatory Research (CBPR) partnership, seeks to reduce tobacco-related health disparities in underserved urban communities. Supported by the National Institute on Minority Health and Health Disparities (NIMHD), CEASE has engaged over 6,000 individuals, developed school-based prevention initiatives, and tested community-based smoking cessation interventions across multiple phases [17–20]. The first three phases demonstrated the program’s effectiveness in various settings, from health clinics to community venues, utilizing a 12-week curriculum. The current phase explores a condensed curriculum to enhance feasibility and accessibility [19,24]. Through collaboration with diverse community partners, CEASE has developed culturally relevant interventions tailored to the needs of urban, low-income populations.

Central to the success of tobacco cessation programs is the reduction in tobacco addiction severity, a critical marker of progress in quitting efforts [37–40]. Tobacco addiction severity can be assessed using various instruments, with the Fagerstrom Test for Nicotine Dependence (FTND) being one of the most widely used tools for evaluating nicotine dependence in adult smokers [41]. The FTND is a reliable measure that predicts several key smoking-related outcomes, including the likelihood of quitting and the risk of relapse [42]. As such, it serves as a valuable metric for assessing the effectiveness of smoking cessation interventions [43]. Lower FTND scores indicate reduced nicotine dependence, which not only increases the likelihood of successful quitting but also supports sustained long-term abstinence [44]. Moreover, reducing nicotine dependence is an important harm reduction strategy. Even if complete cessation is not achieved, lower dependence levels are associated with decreased cigarette consumption, which reduces exposure to smoking-related health risks [45]. Thus, FTND is not only an indicator of intervention efficacy but also a pathway to improved health outcomes [46]. Beyond its use as an evaluation tool, FTND offers an opportunity to investigate whether changes in FTND fully explain the effects of cessation programs on outcomes such as quitting. Testing the concordance between various outcomes allows for a deeper understanding of the nuances on how this tobacco cessation intervention achieve its effects, which may provide additional insights that can guide the design and enhancement of future programs [47–50].

This study builds on previous iterations of the CEASE program by examining whether reductions in tobacco addiction severity, as measured by FTND, can partially or fully explain the effectiveness of CEASE smoking cessation interventions compared to a self-help approach. To understand the validity of the self-reported successful quit in this context, we specifically investigated whether change in FTND fully explains the intervention success. We hypothesize that reduction in FTND scores fully explains the differences between the CEASE intervention vs. the self-help group in self—reported quit rate. This result would indicate that self-report quit is a valid outcome measure.

## 2. Methods

### 2.1. Study Design and Setting

A randomized cluster trial design was used to allocate two underserved Baltimore City communities to one of the two intervention arms of the study: (1) intervention or (2) self-help (control) group. Randomization was blinded and conducted during a steering committee meeting. The Oldtown/Middle East community was randomized to the intervention, while Southwest (included neighborhoods such as Poppleton, Hollins Market, and Washington Village/Pigtown) was assigned to the self-help arm. The study communities were selected based on their similar and comparable sociodemographic characteristics and neighborhood resources, such as schools, healthcare facilities, and faith-based organizations. To enhance recruitment efforts, we later expanded the boundaries of the study communities and enrolled individuals from outside the original study site boundaries, randomizing them based on their recruitment site.

### 2.2. Ethical Considerations

The study underwent a full Institutional Board Review (IRB) and approved by the Morgan State University’s IRB ((IRB #19/06-0082). Each participant signed an informed consent form before enrollment. To maintain confidentiality, each participant was assigned a unique identification number, which was stored independently and securely from their personal information.

### 2.3. Study Participants

The inclusion criteria for this study were adults who were 21 years or older, current smokers (consuming three or more cigarettes per day), willing and ready to quit smoking, and who provided informed consent for participation. Individuals with any health conditions refraining them from providing informed consent were excluded from the study.

### 2.4. Intervention (In-person)

#### 2.4.1. Peer Motivators

To conduct and facilitate the tobacco cessation classes, nine peer-motivators were recruited who were either former smokers (not smoking for at least 12 months) or individuals with a personal connection to tobacco use, such as family members of smokers. The peer-motivators were trained on CEASE Digital curriculum, group facilitation, digital platform integration, research ethics, study procedures and protocols, and data safety and management.

#### 2.4.2. Participant Recruitment

Participants were recruited through community outreach, referrals, flyers, word of mouth, social media, and community surveys. Smoking cessation classes were conducted at local venues, including churches, community centers, senior residences, and public housing sites. Site coordinators at each venue acted as points of contact.

#### 2.4.3. Intervention Arm

The CEASE Today Tobacco Cessation Manual, along with printed educational materials, was used for the intervention which was a seven-week program for each cohort. The first and second weeks consisted of orientation sessions, which included the informed consent process, program orientation, and training on digital device usage. From weeks three to seven, the core smoking cessation curriculum was delivered weekly, emphasizing motivation building during weeks three and four, quitting strategies in weeks five and six, and relapse prevention in week seven. Each session spanned two hours, with two peer motivators assigned to each cohort.

#### 2.4.4. Self-Help (Control) Arm

Participants in the self-help (control) arm were provided with standard resources to support tobacco cessation. These included a one-hour motivational session, self-help materials such as the CEASE Today Tobacco Cessation Manual, and information about local tobacco cessation services. Unlike the intervention arm, no additional group sessions or peer support were offered to participants in this group.

### 2.5. Questionnaires

Baseline characteristics were collected through a questionnaire on the first day of the smoking cessation class (week 1). This included participants’ sociodemographic characteristics, smoking history, physical and mental health, and perceived social support. Follow-up surveys assessed smoking status approximately three months after intervention completion for the group and five months post-enrollment for the self-help group.

### 2.6. Measures

One outcome variable of the study was quit status at follow up (0=No, 1=Yes). Another outcome variable and the explanatory variable for the other outcome was the FTND score. This was assessed using the using the FTND instrument. Nicotine dependence was assessed using the Fagerstrom Test for Nicotine Dependence (FTND) which is a validated and widely used instrument to measure the addiction severity [25,26]. Scores range from 0 to 10, with higher scores reflecting greater nicotine dependence.

Age, gender (male vs. female), education level (1 = some high school or less, 2 = graduated from high school/GED, 3 = some college, 4 = bachelor’s degree or more), income (1 = less than $25,000, 2 = $25,000 or more), general health (1 = poor, 2 = fair, 3 = good, 4 = excellent), depression, perceived social support, intervention arm, and baseline nicotine dependence served as the predictors of the study. Additional variables, including race (categorized as Black Americans, Whites, and other races), employment status (unemployed vs. employed), marital status (not married vs. married), substance use, perceived stress, and class satisfaction, were collected and described but were not included in the analytical models.

Depression was assessed using the Patient Health Questionnaire 2 (PHQ-2), a reliable and validated tool for measuring depression [30]. Participants rated each item based on the frequency of symptoms experienced in the past two weeks, using a scale of 0 (not at all), 1 (several days), 2 (more than half the days), and 3 (nearly every day). The scores from the two items were summed and used as a continuous variable in the SEMs. The Cronbach’s alpha for the items was 0.88, indicating strong internal consistency.

Perceived social support was measured using the 8-Item Duke/UNC Functional Social Support Questionnaire (DUFSSQ) [31]. Participants rated each item on a 5-point Likert scale ranging from 1 (Much less than you would like) to 5 (As much as you would like). The mean score of the eight items was calculated and used as a continuous variable. The scale demonstrated strong internal consistency, with a Cronbach’s alpha of 0.91.

Perceived stress was assessed using the 4-item Perceived Stress Scale (PSS-4) [29]. Each item was measured using a 5-point Likert scale which ranged from 0 (Never) to 4 (Very often). The four items were summed to create a total score ranging from 0 to 16. The Cronbach’s alpha for the items was 0.51.

CEASE class satisfaction was assessed by asking participants, “How satisfied are you with the peer motivation you received?” Responses were recorded on a scale of 1 to 10, where 1 indicated “not at all satisfied” and 10 indicated “extremely satisfied.” Similarly, the helpfulness of the CEASE class was measured with the question, “On a scale of 1 to 10 (1 = not at all useful and 10 = extremely useful), how useful were the CEASE classes based on your experience?” Both variables were treated as continuous measures in the descriptive analysis.

### 2.7. Statistical Analysis

Univariate analysis was conducted, with results reported as means with standard deviations (SD) and proportions. Bivariate analysis was performed to examine the relationships between predictors and intervention arms. Chi-square tests were used for categorical variables, while Kruskal-Wallis tests were employed for continuous variables. We used regression analysis to test the association between intervention, outcomes, and covariates. We also ran two structural equation models (SEMs) to investigate whether FTND explains the follow-up change in self-rated quit rate on smoking cessation. *Model 1* did not include the explanatory variable (FTND score at baseline), while *Model 2* included this variable. We reported standardized coefficients (β), along with their standard error (SE), and significant *p*-values for each model. Significance level was set at p < 0.05. Stata version 15.0 (StataCorp LLP) was used for the analysis.

## 3. Results

### 3.1. Descriptive Results

This study analyzed data from 142 current smoker adults who completed the follow-up for our two-arm intervention. Participant characteristics are summarized in Table 1. The mean (SD) age of the participants was 54.9 (11.9) years. The majority of the participants were men (50.7%) and Black Americans (78.2%). Approximately 35.2% of the participants graduated from the high school or completed a GED, 56.3% had an income of less than $25,000, and 85.9% were unemployed.

The two study arms showed no significant differences in education level, family income, employment status, marital status, depression scores, perceived stress, perceived social support, or baseline nicotine dependence. However, the groups differed in racial composition, gender, and nicotine dependence at follow-up. The self-help group had a higher proportion of men (66.7%) and White participants (23.8%) compared to the group, which had 38.0% men and 10.1% White participants. Additionally, the self-help group had a higher mean (SD) Fagerstrom score for nicotine dependence at follow-up, at 3.9 (2.6), compared to 2.6 (2.1) for the group.

**Figure 1.**
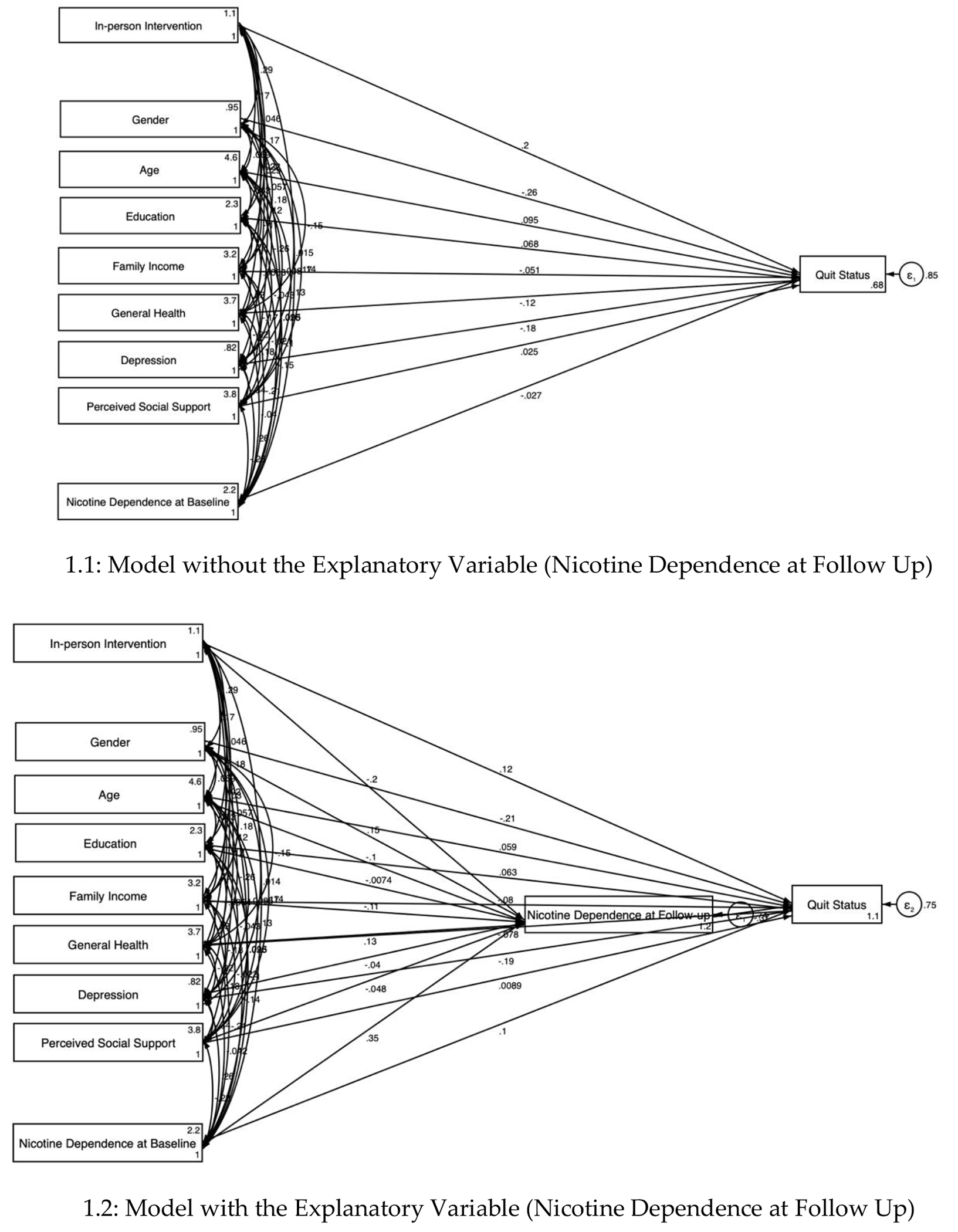
Structural Equation Model

Table 2 presents the SEM results (Figure 1). *Model 1*, which did not include the mediator, showed that intervention had positive association (B = 0.20, p < 0.05) with quitting smoking. Being a woman (B = −0.26, p < 0.01) and having higher depression score (B = −0.18, p < 0.05) were negatively associated with quitting smoking.

*Model 2*, which included the mediator (nicotine dependence at follow-up), revealed a strong and significant negative association (B = −0.37, p < 0.001) between nicotine dependence at follow-up and quitting smoking. The effects size decreased and became nonsignificant for the association between intervention and quit status (B = 0.12), suggesting that nicotine dependence at follow-up mediates the relationship between intervention and quitting smoking. Although the negative association between being a woman and quitting smoking weakened in Model 2, it remained significant (B = −0.21, p < 0.05). A higher depression score also showed negative association (B = −0.19, p < 0.05) with quitting smoking. The intervention was negatively associated with nicotine dependence at follow-up (Beta = − 0.20, p < 0.05).

Both *Model 1* and *Model 2* showed an excellent fit to the data, as evidenced by the Root Mean Square Error of Approximation (RMSEA) of 0.000, a Comparative Fit Index (CFI) of 1.000, and a Tucker-Lewis Index (TLI) of 1.000. These results indicated well-specified model with strong fit indices.

## 4. Discussion

This study provides important insights into the validity of self-reported quit in the CEASE smoking cessation intervention among underserved populations, particularly low-income Black adults. Our findings highlight that the reduction in tobacco addiction severity, as measured by the FTND, is in line with the self-reported quit in the CEASE intervention program. Participants who self-reported smoking cessation demonstrated significantly greater reductions in FTND scores compared to their peers, and the FTND reduction fully explained the intervention’s impact on self-reported quit rate.

The results indicate that the effectiveness of the CEASE intervention measured based on self-reported successful quit is entirely in line with a reduction in nicotine dependence (FTND score). This aligns with existing literature emphasizing the role of addiction severity as a critical marker of progress in quitting efforts [37–40]. The FTND, widely recognized for its reliability and validity, was instrumental in measuring addiction severity in this study [41]. This tool is particularly relevant for interventions targeting underserved populations, as it predicts key smoking-related outcomes, such as the likelihood of quitting and the risk of relapse [42]. Lower FTND scores facilitate quitting and support sustained abstinence, underscoring the importance of targeting nicotine dependence as part of smoking cessation efforts [43]. Furthermore, reducing addiction severity serves as a harm reduction strategy, as individuals with lower dependence often smoke fewer cigarettes, reducing their exposure to smoking-related health risks even if complete cessation is not achieved [44]. By demonstrating that changes in FTND scores explain the intervention’s effects, this study advances our understanding of the mechanisms underlying successful smoking cessation programs [45].

Our findings also reinforce the value of interventions in reducing tobacco addiction severity, particularly for low-income Black populations who face unique structural barriers. Direct, face-to-face interaction in the CEASE program likely provided real-time engagement, peer motivation, and immediate emotional support, all of which are critical in fostering accountability and behavior change [46–50]. These interpersonal dynamics are integral to the success of high-touch, community-based interventions, especially for populations that experience significant health inequities related to smoking cessation.

This study’s focus on low-income, predominantly Black participants highlights the persistent structural barriers that hinder smoking cessation in underserved communities. Challenges such as unemployment, low educational attainment, and chronic stress can reduce the effectiveness of interventions by exacerbating addiction severity and limiting participants’ capacity to engage fully with cessation programs. Addressing these structural inequities is essential for improving outcomes in these populations.

The findings also underscore the importance of tailoring public health strategies to meet the specific needs of marginalized communities. While virtual interventions offer scalability and convenience, their utility was not the focus of this study, which showed that approaches remain highly effective for low-income populations. Future interventions should prioritize high-touch, community-driven models that incorporate culturally relevant content, peer support, and local resources to maximize their impact.

### 4.1. Limitations

Several limitations should be considered when interpreting these results. First, participants were recruited based on their willingness to quit smoking, potentially introducing selection bias and limiting the generalizability of the findings. Second, the study was conducted in specific low-income communities in Baltimore, which may not reflect the experiences of other populations or regions. Additionally, self-reported measures of smoking behavior could result in inaccuracies when assessing reductions in tobacco addiction. Finally, while the study controlled for several key variables, unmeasured confounders, such as mental health status or prior quit attempts, may have influenced the outcomes.

## 5. Conclusion

There is concordance in measuring the effects of the CEASE program using self-reported outcome, or a more objective FTND score. In fact, change in the FTND score fully explains (validates) the CEASE intervention effect on self-reported outcome (quit). These findings suggest that CEASE is an effective program for underserved populations who use tobacco.

## Figures and Tables

**Table 1.** Bivariate Analyses Comparing Participants between the Two Arms

Variables	In-Person CEASE (n= 79)	Self-help/Control Group (n=63)	Total (n=142)

	n (%)	n (%)	n (%)

Gender**			
Men	30 (38.0)	42 (66.7)	72 (50.7)
Women	45 (56.9)	19 (30.1)	64 (45.1)
Missing	4 (5.1)	2 (3.2)	6 (4.2)
Race			
Black American	68 (86.1)	43 (68.2)	111 (78.2)
White	8 (10.1)	15 (23.8)	23 (16.2)
Other/Multiple	2 (2.5)	3 (4.8)	5 (3.5)
Missing	1 (1.3)	2 (3.2)	3 (2.1)
Educational Attainment			
Some high school or less	19 (24.1)	18 (28.6)	37 (26.1)
Graduated from high school/GED	29 (36.7)	21 (33.3)	50 (35.2)
Some college	20 (25.3)	14 (22.2)	34 (23.9)
Bachelor or more	11 (13.9)	8 (12.7)	19 (13.4)
Missing	-	2 (3.2)	2 (1.4)
Family Income			
Less than $25,000	40 (50.6)	40 (63.5)	80 (56.3)
$25,000 or more	11 (13.9)	3 (4.8)	14 (9.9)
Missing	28 (35.5)	20 (31.7)	48 (33.8)
Employment			
No	68 (86.08)	54 (85.71)	122 (85.9)
Yes	10 (12.66)	6 (9.52)	16 (11.3)
Missing	1 (1.27)	3 (4.76)	4 (2.8)
Marital Status			
Not married	71 (89.9)	49 (77.8)	120 (84.5)
Married	8 (10.1)	12 (19.0)	20 (14.1)
Missing	-	2 (3.2)	2 (1.4)
General Health			
Poor	2 (2.5)	4 (6.4)	6 (4.2)
Fair	36 (45.6)	28 (44.4)	64 (45.1)
Good	37 (46.8)	23 (36.5)	60 (42.3)
Excellent	4 (5.1)	6 (9.5)	10 (7.0)
Missing	-	2 (3.2)	2 (1.4)
	Mean (SD)	Mean (SD)	Mean (SD)
Age (years)***	56.7 (13.4)	52.7 (9.4)	54.9 (11.9)
Nicotine Dependence (at baseline)	4.1 (1.9)	4.8 (2.0)	4.4 (2.0)
Nicotine Dependence (at follow-up)**	2.6 (2.1)	3.9 (2.6)	3.2 (2.4)
Depression	1.4 (1.9)	1.6 (1.7)	1.5 (1.8)
Perceived Social Support	4.1 (1.0)	3.8 (1.1)	4.0 (1.1)

Abbreviations: SD= Standard Deviation;

*** p < 0.001,

** p < 0.01,

* p < 0.05.

**Table 2.** Summary of the SEM Result

Variables				
	*Model 1* (Without Explanatory Variable)		*Model 2* (With Explanatory Variable)	

	B	SE	B	SE
Outcome: Quit Status				
Intervention	0.20*	0.08	0.12	0.08
Nicotine Dependence at Follow-up	NA	NA	−0.37***	0.08
Nicotine Dependence at Baseline			0.10	0.09
Age (years)	0.10	0.08	0.06	0.08
Gender (Female)	−0.26**	0.09	−0.21*	0.08
Education (1-4)	0.07	0.08	0.06	0.08
Household Income (1-5)	−0.05	0.10	−0.08	0.10
General Health	−0.12	0.09	−0.08	0.08
Depression	−0.18*	0.09	−0.19*	0.08
Perceived Social Support	0.02	0.09	0.01	0.09

Outcome: Nicotine Dependence at Follow-up				
Intervention	NA	NA	−0.20*	0.08
Nicotine Dependence at Baseline	NA	NA	0.35***	0.08
Age (years)	NA	NA	−0.10	0.08
Gender (Female)	NA	NA	0.15	0.08
Education (1-4)	NA	NA	−0.01	0.08
Household Income (1-5)	NA	NA	−0.11	0.10
General Health	NA	NA	0.13	0.08
Depression	NA	NA	−0.04	0.08
Perceived Social Support	NA	NA	−0.05	0.09

Note B: Standardized Coefficient; SE= Standard Error; NA: Not Applicable.

*** p < 0.001,

** p < 0.01,

* p < 0.05.
